# Supplementary material for: Effects of Abelmoschus manihot Flower Extract on Enhancing Sexual Arousal and Reproductive Performance in Zebrafish
Source: Molecules. 2022 Mar 29;27(7):2218. doi: 10.3390/molecules27072218 (PMC9000255; doi:10.3390/molecules27072218)
Supplement: Supplementary file 1 [file molecules-27-02218-s001.zip › molecules-1650301-supplementary.pdf]

GGATCATTGTCGAAGCCTGCCCAGCAGAACGACCCGCGAACGTGTTATCGAAAAACAACGG
GACGGGCGAGGCGGGATCCCCGCCCCCCGTCCCACCCCGCCCCGGTGCCCCTCGCCGTCGC
CTCCCCCTCGCCTCACGGTGCCGCGGGATGCACGGCCCCGGGCCTCCGGGGCGAAACGAAC
AACCCCCGGCGCGAATCGCGCCAAGGAACCTGAATTGAAAGGAGCACGTCCCCCGTCGCCG
CCCCGTCCGCGGTGCGCGTGCTGCGGGGACGCTGCGACTTCGTCGTGAATACACAAAACGA
CTCTCGGCAACGGATATCTCGGCTCTCGCATCGATGAAGAACGTAGCGAAATGCGATACTT
GGTGTGAATTGCAGAATCCCGTGAACCATCGAGTCTTTGAACGCAAGTTGCGCCCCAAGCC
GTCAGGCCGAGGGCACGTCTGCCTGGGTGTCACGCATCGTCGCTCCCATCCAACCCCTCCC
CCCGGGGACGGGCTGCGGTGTGGGCGGACAATGGCCTCCCGTTCGCACACCGCTCGCGGTT
GGCCCAAAATCGAGTCATCGGCGACCACGGTGCCGCGACGATCGGTGGTAACGCTTCGAGC
TGCCTCTTTCGTAGTCGCGCGCCAACGTCGTCCCCGGCTCCCCGACCCTTTCGGCACCGCA
AGCACGGTGCCCGCGTCGCGACCCCAGGT (700 bp)

Supplementary Figure S1. The nucleotide sequence of *A. manihot* flower used in this study. The analyzed sequences comprise the partial 18S ribosomal RNA gene, complete internal transcribed spacer 1, 5.8S ribosomal RNA gene, internal transcribed spacer 2, and partial 28S ribosomal RNA gene.
